# Acute Stress Alters Amygdala microRNA miR-135a and miR-124 Expression: Inferences for Corticosteroid Dependent Stress Response

**DOI:** 10.1371/journal.pone.0073385

**Published:** 2013-09-04

**Authors:** Cecilia Mannironi, Jeremy Camon, Francesca De Vito, Antonio Biundo, Maria Egle De Stefano, Irene Persiconi, Irene Bozzoni, Paola Fragapane, Andrea Mele, Carlo Presutti

**Affiliations:** 1 Institute of Cellular Biology and Neurobiology, Consiglio Nazionale delle Ricerche, A. Buzzati-Traverso Campus, Monterotondo, Rome, Italy; 2 Department of Biology and Biotechnology Charles Darwin, Sapienza University of Rome, Rome, Italy; 3 Center for Research in Neurobiology, Sapienza University of Rome, Rome, Italy; 4 Institute Pasteur Fondazione Cenci-Bolognetti, Rome, Italy; 5 Institute of Molecular Biology and Pathology, Consiglio Nazionale delle Ricerche, Sapienza University of Rome, Rome, Italy; CNRS UMR7275, France

## Abstract

The amygdala is a brain structure considered a key node for the regulation of neuroendocrine stress response. Stress-induced response in amygdala is accomplished through neurotransmitter activation and an alteration of gene expression. MicroRNAs (miRNAs) are important regulators of gene expression in the nervous system and are very well suited effectors of stress response for their ability to reversibly silence specific mRNAs. In order to study how acute stress affects miRNAs expression in amygdala we analyzed the miRNA profile after two hours of mouse restraint, by microarray analysis and reverse transcription real time PCR. We found that miR-135a and miR-124 were negatively regulated. Among *in silico* predicted targets we identified the mineralocorticoid receptor (MR) as a target of both miR-135a and miR-124. Luciferase experiments and endogenous protein expression analysis upon miRNA upregulation and inhibition allowed us to demonstrate that mir-135a and mir-124 are able to negatively affect the expression of the MR. The increased levels of the amygdala MR protein after two hours of restraint, that we analyzed by western blot, negatively correlate with miR-135a and miR-124 expression. These findings point to a role of miR-135a and miR-124 in acute stress as regulators of the MR, an important effector of early stress response.

## Introduction

Stress can be broadly defined as a disruption of homeostasis, to which the organism responds by trying to re-establish the initial equilibrium or to adopt an altered state in the new environment. The adaptive response to homeostatic disturbances implies that the stress response is activated rapidly and terminated efficiently afterwards. If coping with stress fails, a vulnerable phenotype with increased susceptibility to psychopathologies is produced [1]. Stressor-related information from all sensory systems are conveyed to a variety of limbic brain structures, as hippocampus, amygdala and prefrontal cortex, that work in parallel but are involved in different aspects of the stress response [2,3]. In particular the amygdala, a group of nuclei located in the medial temporal lobe, is considered a key node for stress response integration, with a widespread network of efferent projections to other brain regions [4,5]. Stress mediators, as (nor) adrenaline, corticotrophin releasing hormone (CRH) and corticosteroids (CORT; corticosterone in rodents, cortisol in humans), are highly conserved among vertebrates and contribute to the neuronal functional change and plasticity that are instrumental to the stress response [6]. Acute psychological stress causes a rapid surge of neurotransmission, neuronal activation and hormone release. Though temporary, this activation has profound effects in the brain, ultimately leading to altered gene expression and structural modifications in dendritic spine morphology and synaptic connectivity [1]. This stress-induced neuronal plasticity is responsible for changing the subsequent neuronal response, and shows unique features in the amygdala [7].

miRNAs are a growing class of small non-coding RNAs that act as post-transcriptional regulators of gene expression primarily by translational repression [8,9]. In mammals, they are implicated in the control of many fundamental processes and most of them are expressed in a development- or tissue-specific manner [8,10,11]. The nervous system is a rich source of miRNAs [10–12], consistent with their primary role in brain development and neuronal cell identity maintenance [13–15]. More recently miRNA functions in the mature nervous system have been related to neuronal plasticity and to the control of synaptic tasks [16].

The ability of miRNAs to selectively [17] and reversibly [18] silence mRNAs, together with their involvement in neuronal plasticity events, make miRNAs well-suited to serve as fine regulators of the complex and extensive molecular network involved in stress response. In this report we investigated whether in the amygdala the process of acute stress response involves miRNAs. We show that after two hours of mouse restraint miR-135a and miR-124 are negatively regulated. Demonstrating that miR-135a and miR-124 are able to affect the MR expression, we suggest their functional role in the initial stress reaction by the activation of the corticosteroid signaling.

## Materials and Methods

### Ethics statement

All animals were housed, cared for, and experiments conducted in accordance with the guidelines laid down by the European Community Council Directive (86/609/EEC of 24 November 1986), the Italian National Low guidelines and the National Institutes of Health Guide for the Care and Use of Laboratory Animals. This study was approved by the Italian Department of Health (authorization D.M. n° 169/2009-B to AM).

### Animals and stress procedures

Adult male C57BL/6J/Cnrm mice 13-15 weeks old were used for stress experiments. Mice were kindly provided by the European Mouse Mutant Archive, Consiglio Nazionale delle Ricerche (CNR-EMMA, Monterotondo, Italy). All mice were allowed to acclimate to the colony for at least four weeks before handling, they were kept on a 12 hours light/dark cycle, with food and water *ad libitum*. Mice were randomly assigned to two different groups: acute stress mice, subjected to a single 2 hour restraint session in a black perforated plastic tube (diameter: 3cm, length: 10cm); naive mice, briefly handled in a separate room. All stress sessions were done between 10:00 a.m. and 4:00 p.m. Immediately after the stress or handling session, the animals were sacrificed, and tissues were dissected, flash frozen and stored at -80°C. Amygdala punches were obtained with a 1-mm punch tool.

### Corticosterone measurement

Immediately after acute immobilization stress, blood was collected and stored in EDTA containing tubes. To obtain plasma the tubes were centrifuge at 10000 rpm for 10 min and kept at -80°C until use. Concentration of corticosterone in plasma was quantified by ELISA (Cayman Chemical Company) according to the instructions of the manufacturer.

### RNA extraction and quantitative RT-PCR

Total RNA was isolated from cells and from dissected brain tissue according to the standard Trizol (Life Technologies) protocol, with one additional extraction with chloroform before precipitation in 3 volume of ethanol. The tissue was homogenized in Trizol with a Dounce homogenizer prior to extraction. The quantity and the quality were analyzed on a NanoDrop 1000 spectrophotometer (Thermo, Fisher Scientific) and by visual inspection of the agarose gel electrophoresis images. RNA was extracted from single animals and RNA pools were produced. Quantitative Reverse Transcription-PCR (qRT-PCR) analysis of miRNAs was done by TaqMan miRNA assay (Applied Biosystems, Life Technologies) according to the manufacturer’s protocol. qRT-PCR analysis of Nr3c2 mRNA was done on cDNAs prepared using SuperScript III (Life Technologies) and dT primers plus random primers at 50°C for 2 hours. qPCR was performed using Syber-Green (SensiMix^TM^ SYBR Hi-ROX Kit, Bioline) with appropriate primers (See Supplementary Methods for primer sequences). Relative quantification of gene expression was conducted with the Applied Biosystems 7300 Real Time PCR System and data analysis was performed using the comparative ΔΔC_T_ method. U6B and sno202 RNAs were used as internal control for miRNA expression, Actin mRNA as internal control for mRNA expression.

### microRNA array profiling

2.5µg of total amygdala RNA from pooled nuclei (*n*=12) from naive and stressed mice were Hy3- and Hy5-labeled respectively, using miRCURY^TM^ LNA microRNA Array Power Labeling Kit (Exiqon). Fluorochrome-labeled RNA samples were then combined, denatured and hybridized to custom-made slides containing LNA-modified microRNA capture probes targeting all human, mouse and rat miRNAs listed in the miRBase Sequence Database Release v.11.0 (http://microrna.sanger.ac.uk/sequences/), which contains 1769 unique mature miRNA sequences. Slides have been kindly provided by Dr. V. Benes, from the Genomics Core Facility of the European Molecular Biology Laboratory (Heidelberg, Germany). The hybridization was performed according to the miRCURY^TM^ LNA array manual using hybridization chambers (Agilent), for 16 hours at 56°C. After hybridization, the microarray slides were scanned using a ScanArray Lite Microarray Scanner (Packard Bioscience) and the image analysis was carried out using GenePix® Pro 7 Software (Molecular Devices). Absent and marginal spots were flagged automatically by the software, and then each slide was inspected manually. Data were normalized using different endogenous controls present in the LNA-modified spotted library. The Hy5/ Hy3 ratios were log_2_-transformed. Data from 2 independent experiments were averaged and only probes with a log_2_-ratio above 1 or below -1 were considered. Only probes with log intensity >8 and <14 were taken in account, to avoid non linear effects caused by the noise floor at low intensities or by saturation at high [19].

### DNA constructs

miRNA expression vector p135a was obtained by amplifying 301-bp from mmu-miR-135a-1 genomic sequence, containing 111 nt upstream and 100 nt downstream the miRNA stem-loop sequence, according to the miRBase Database (http://www.mirbase.org/). The PCR fragment was cloned dowstream the U1 promoter of a pSP65 vector [20]. The complete Nr3c2 3’ UTR was amplified from mouse genomic DNA and cloned into the XbaI unique site of the pGL3 control vector (Promega). To generate Nr3c2 mutant constructs Nr3c2 m135a and Nr3c2 m124, mutations of seed binding sites for miR-135a (Nr3c2 m135a) or miR-124 (Nr3c2 m124) were introduced into the Nr3c2 3’ UTR using synthetic oligonucleotides, by generating partially complementary PCR fragments. These fragments were used as templates for PCR to generate complete mutated Nr3c2 3’ UTR fragments, further cloned as described for wild type 3’ UTR. All constructs have been checked by sequencing. See Supplementary Methods for primer sequences.

### Cell culture and transfection

Hela cells and Neuro-2a (N2a) mouse neuroblastoma cells were obtained from ATCC (http://www.lgcstandards-atcc.org/). Cells were cultured in DMEM (Life Technologies) plus 10% FBS, 1 mM L-glutamine, 100 U ml^-1^ penicillin, and 100 µg ml^-1^ streptomycin. Transfections were performed at 70% confluency using Lipofectamine 2000 (Life Technologies), according to the manufacturer’s protocol.

Primary cultures of cerebellar granule neurons (CGN) were prepared from cerebella of C57BL/10 mice at postnatal day 8 (P8), according to established protocols [21]. Briefly, mice were deeply anesthetized with Isofluorane-Vet (isofluorane, Merial) and decapitated; brains were quickly removed and collected in ice-cold HBSS solution, pH 7.3 (3 mM HEPES, 1% penicillin-streptomycin and 1X Hank’s Buffered Salt Solution). Cerebella were rapidly dissected in the same solution and cut into small pieces after having carefully removed the meninges. Tissue pieces were incubated for 15 min at 37°C in a digestion buffer (0.1% trypsin, 0.25 mg/ml DNAse in PBS) and successively triturated through a flame-polished Pasteur pipette until no chunks were visible. Cells were centrifuged for 10 min at 1000 rpm (4°C), re-suspended in DMEM culture medium (2 mM glutamine, 2% B27, 1% penicillin-streptomycin, 5% fetal bovine serum, 5 mM D-glucose in DMEM-Dulbecco’s modiﬁed Eagle’s medium), counted in an hemacytometer and plated at a density of 10^6^ cells/35 mm in Petri dishes, previously coated with 0.1 mg/ml polylysine. Glial cells proliferation was inhibited by adding to the culture medium, 18-22 hours after plating, 10 mM of cytosine-beta-D-arabinofuranoside (Ara-C). After 6 days, Ara-C was removed and cells maintained *in vitro* up to 10 days (10 DIV).

### Luciferase assays

Hela cells were transfected in 24-well plates with 0.2 µg of pGL3 constructs, 0.8 µg of miRNA expression vectors, and 0.02 µg of pRL vector. The Renilla expressing pRL vector was used as an internal control. Cells were harvested 24 hours after transfection and luciferase activities were measured using the Dual-Luciferase Reporter Assay System (Promega) as described by the manufacturer’s protocol.

### Western Blot analysis

For endogenous protein expression analysis, N2a cells and CGN (6 DIV) were transfected in 6-well plates with 4 µg of miRNA expression vectors or 100 nM LNA (scramble, LNA anti miR-135a or LNA anti miR-124 (Exiqon), respectively). Cell lysates were prepared 72 hours after plasmid and 96 hours after LNA transfections. Protein extracts were obtained using RIPA lysis buffer (150 mM NaCl, 50 mM Tris pH8.0, 0.5% sodium deoxycholate, 0.1% SDS, 1% Nonidet P-40) containing Complete Protease Inhibitor Cocktail (Roche). Total protein extracts from mouse tissues were obtained from pools of amygdala nuclei (*n*=4) by homogenizing in RIPA buffer. Proteins were separated by SDS-PAGE and blotted onto a nitrocellulose membrane (Whatman). Non-specific bindings were blocked with Tris-buffered saline plus 5% milk powder and 0.2% Tween 20. The following primary antibodies were used: mouse monoclonal anti-MR (1:200; antibody raised against the rat MR peptide epitope AA64-82) [22]; rabbit anti-actin (1:2000; A2066; Sigma Aldrich); mouse anti-tubulin (1:5000; T3526; Sigma Aldrich). The secondary horseradish peroxidase (HRP)-conjugated goat anti-rabbit antibody (1:20000; A0545; Sigma Aldrich) or anti-mouse antibody (1:20000; A5278; Sigma Aldrich) were visualized by enhanced chemiluminescence with a western blotting detection kit (Bio-Rad) according to the manufacturer’s instructions. Band intensities were quantified by densitometric analysis. For quantitative analysis of WB the signal for each protein was normalized to the housekeeping protein detected on the same blot, and the ratio with average control values was determined.

### Bioinformatic analysis

Prediction analysis of the miR-135a targets was done using three prediction algorithms, microT v 3.0 (diana.cslab.ece.ntua.gr/microT), TargetScan 5.2 (www.targetscan.org), and PicTar (www.pictar.mdc-berlin.de). Candidate genes identified by all three algorithms were analyzed by DAVID 6.7 (http://david.abcc.ncifcrf.gov/) for functional annotation and clustering, tissue expression and pathway assignment [23–25]. Identification of miRNAs targeting mouse Nr3c2 3’ UTR was done by microT v 3.0, PicTar and TargetScan 5.2.

### Statistical analysis

Statistical significance was evaluated using Student’s *t*-test, or one-way ANOVA performed by the StatView software. The probability values less than 5% or 1% were considered significant.

## Results

### Acute stress affects amygdala miRNA expression

**Figure 1:**
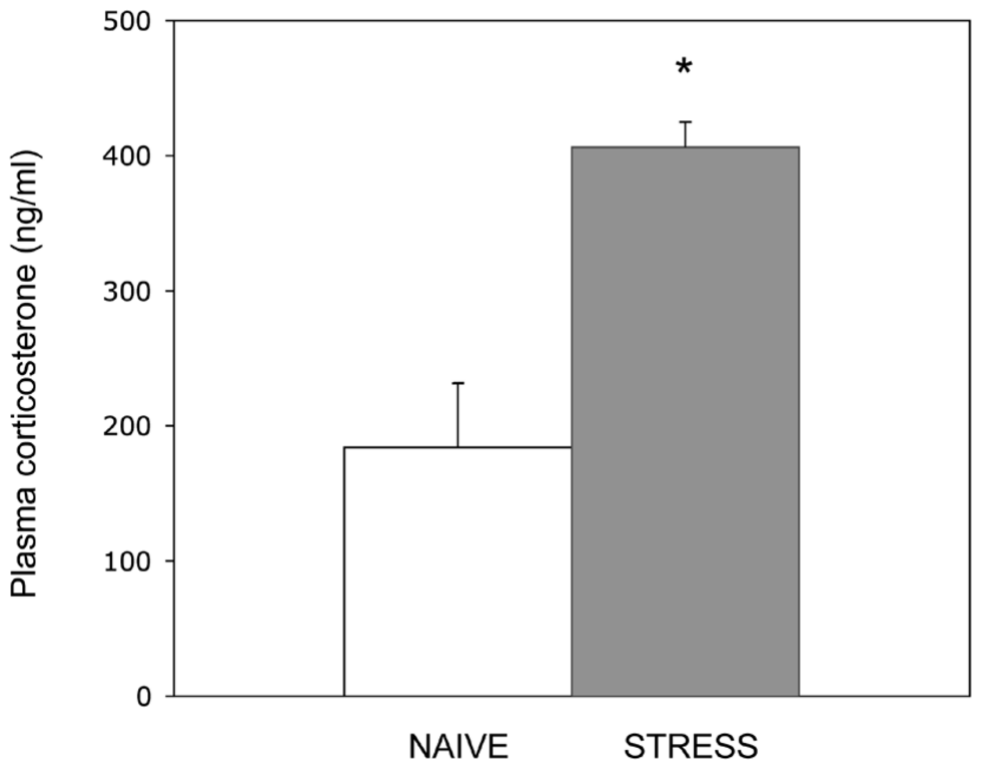
Restraint stress induces an increase of plasma corticosterone levels. Plasma corticosterone levels were measured by ELISA under basal conditions (Naive) and immediately after acute restraint stress (Stress). Bars represent mean ± SE, *P<0.05 (pairwise Student’s *t*-test).

**Figure 2:**
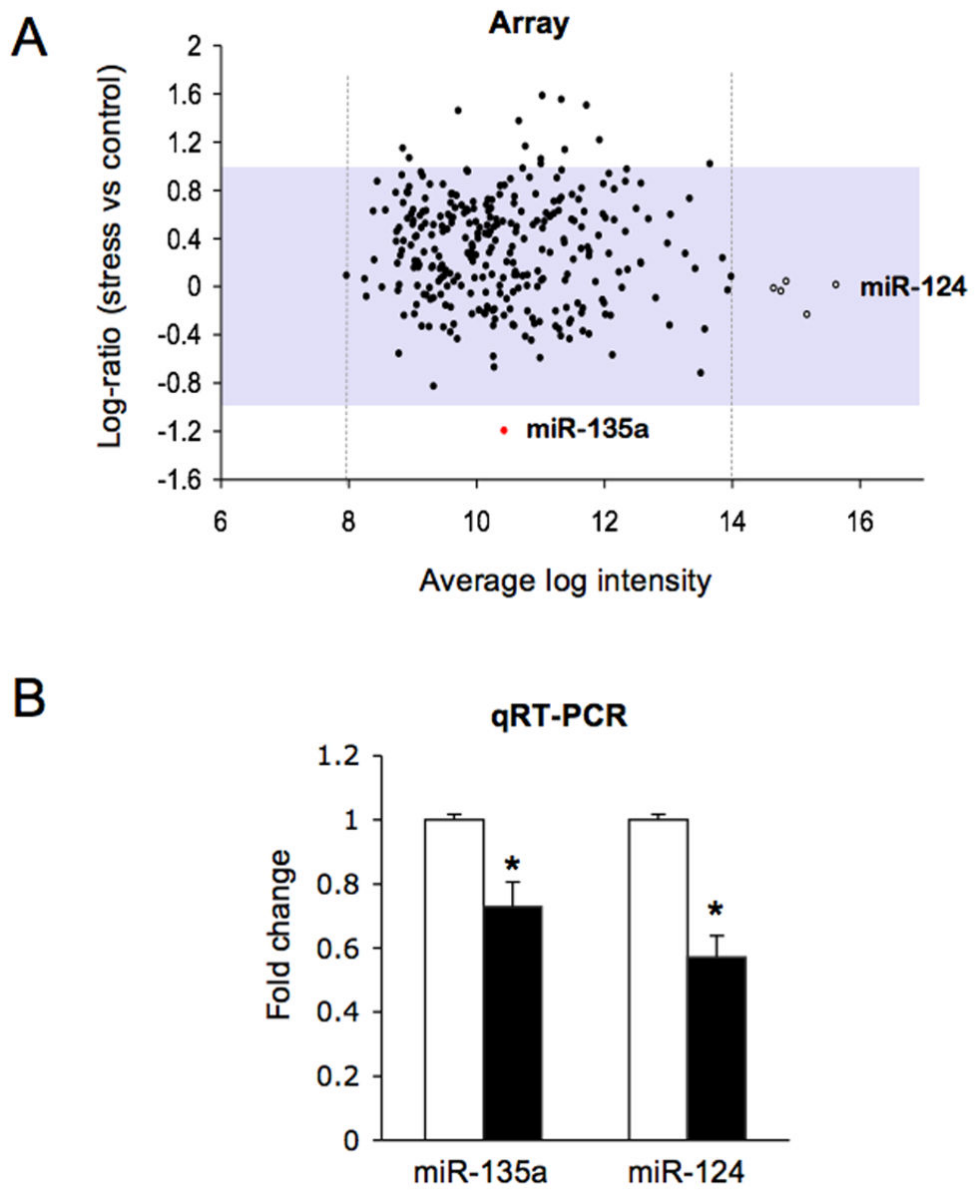
Acute stress induces miR-135a and miR-124 downregulation in the amygdala. (A) The differential expression of mature miRNAs in the amygdala was evaluated by microarray analysis after 2 hours of restraint. The MA plot shows relative change values, expressed as log_2_ ratio (stress vs control), plotted against average log intensity ((log_2_Hy5+log_2_Hy3)/2). (B) Levels of mature miRNAs are quantified in the amygdala RNA pool by qRT-PCR, using U6B as internal control. The statistical test used for comparison was one-way ANOVA (*n*=9). Values are means ± SE *P <0.001 versus naive control mice.

In rodents, a single immobilization session represents a brief but severe stress that triggers in amygdala a surge of corticosterone and glutamate release that eventually leads to synaptic plasticity [5,26]. To examine the neuroendocrine effects of two hours of restraint we measured circulating corticosterone levels immediately after stress, finding a significant increase in plasma corticosterone in restrained compared to naive mice (Figure 1). We examined whether two hours of acute stress altered miRNA expression in the mouse amygdala, by using miRNA microarray profiling as an initial screening approach. Pooled RNA samples from stressed and naive mice (*n*=12) were hybridized to an LNA platform that allowed us to analyze the relative expression of the 288 miRNAs that could be detected in our biological samples. The amygdala miRNA expression profile is altered after acute stress (Figure 2A), indicating an increase of up to three-fold in the expression of several miRNAs (log_2_-ratio=1.6) and a decrease in the expression of a minority of miRNAs. Among miRNAs negatively regulated, only one miRNA shows a log_2_-ratio < 1, namely miR-135a. Its expression level was found to be more than two times lower in stressed compared to naive mice (log_2_-ratio=-1.2). In order to further investigate stress-induced changes of miRNA expression in amygdala, we performed qRT-PCR analysis on a panel of neuronal highly expressed miRNAs, whose signals in our array study were above the signal intensity threshold of 14 (white dots in Figure 2A). Among the miRNAs studied, miR-124 showed significantly reduced levels of about 40% in RNA samples from stressed compared to naive mice (*n*=12) (Figure 2B). Moreover, qRT-PCR analysis indicated an approximately 30% reduction of the expression levels of miR-135a, validating our previous array data (Figure 2B). Similar results were obtained normalizing data with sno202 as internal control (Figure S1). Thus, we conclude that mir-135a and mir-124 expression levels are sensitive to stress, as indicated by qRT-PCR for miR-124, and by both microarray and qRT-PCR for miR-135a.

The expression of miR-124 in the nervous system has been broadly investigated, both in the development and in the adult mouse CNS. It is highly enriched and widely expressed in the mouse brain, with some region specificity [10–15,27]. miR-135a is brain-specific, its expression is induced upon neuronal differentiation, and it is poorly expressed in other adult tissue [10,11,27–29]. However, not much is known about miR-135a expression in the different brain regions. By northern blot analysis we studied miR-135a expression in different structures of the adult mouse brain and in post-natal neurons (Figure S2): various expression levels were found in the brain areas analyzed, including the amygdala, with a higher relative expression in the cerebellum, as already described [30]. A significant miR-135a expression was found in primary neuronal cultures of cerebellar granule neurons.

### miR-135a and miR-124 regulate Nr3c2 mRNA expression

Next, to better understand the possible role of mir-135a and miR-124 downregulation in the context of the stress response, we searched for predicted mRNA targets. Using three independent target prediction algorithms, DIANA-microT v 3.0, TargetScan 5.2, and PicTar, we narrowed the number of bioinformatic target candidates for miR-135a to 38 genes (Figure S3). Interestingly, from our bioinformatic analysis the top-score predicted target is the nuclear receptor subfamily 3, group C, member 2 (Nr3c2), also known as MR, the highest-affinity receptor for corticosteroid hormones in the brain [31]. From now on Nr3c2 refers to the gene and the mRNA, MR to the protein.

**Figure 3:**
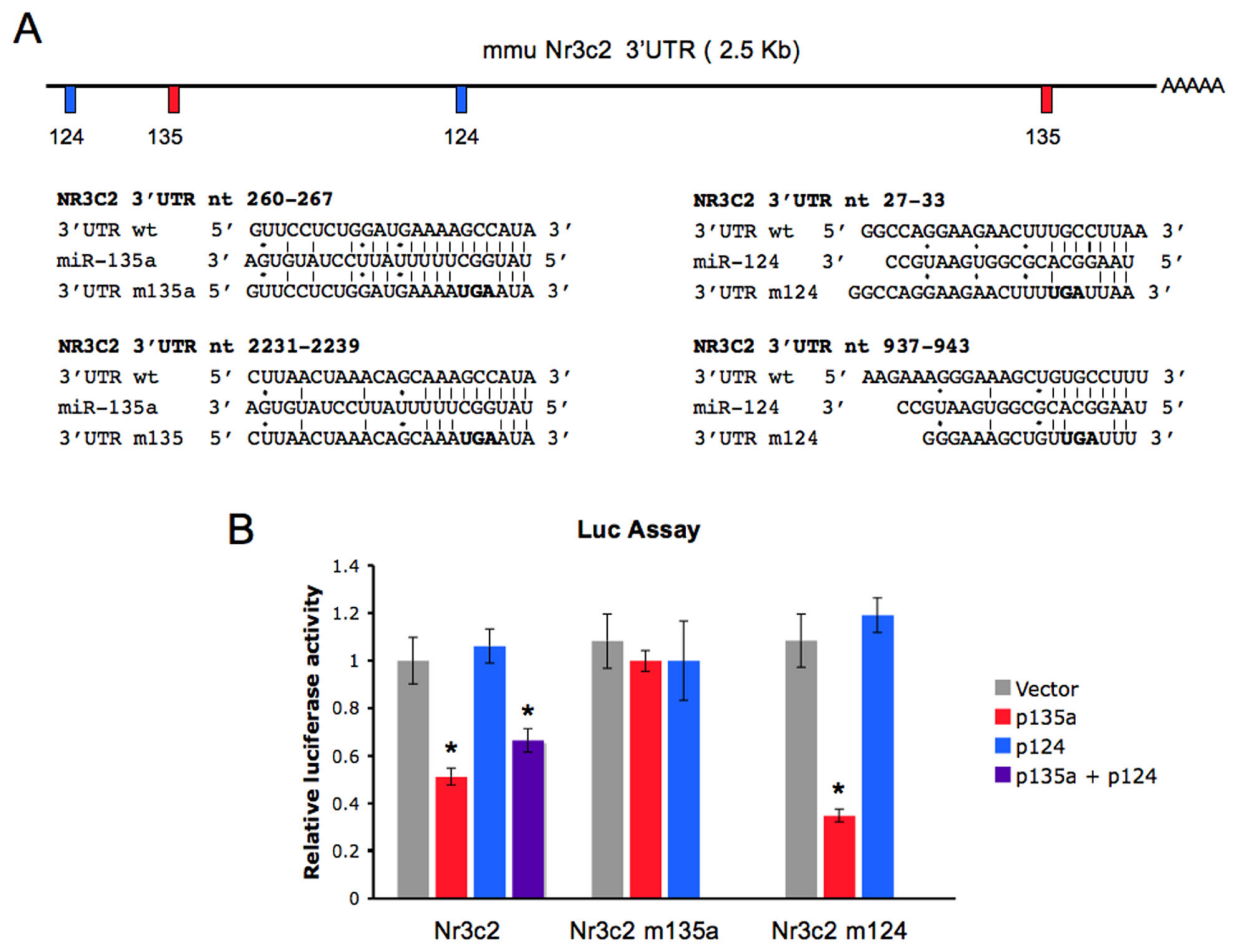
Nr3c2 reporter is regulated by miR-135a. (A) High probability miRNA target sequences in the mouse Nr3c2 3’ UTR are drawn. Candidate miRNAs are predicted by microT v 3.0, TargetScan 5.2, and PicTar algoritms. Positions of mir-135a and miR-124 target sequences in the mouse annotated Nr3c2 3’ UTR and details of miRNA/mRNA base pairing are indicated. Beneath miRNA sequences, nucleotides mutated at the level of miRNA binding sites in the mutant constructs Nr3c2 m135a (mutated at both miR-135a seed binding sites) and Nr3c2 m124 (mutated at both miR-124 seed binding sites), are indicated (nts in bold). (B) Nr3c2 luciferase reporter (Nr3c2), and mutant reporter constructs were co-transfected into Hela cells together with empty vector or miRNA expression vectors (p135a and p124). Luciferase activity was measured 24 hours post-transfection. Values are expressed relatively to the internal renilla luciferase activity and presented as percentage of the activity achieved in the presence of the empty control vector. Results are shown as means ± SE (n=6). **P<0.005 (pairwise Student’s *t*-test).

Two binding sites for miR-135a are present upstream and dowstream in the mouse Nr3c2 3’ UTR (Figure 3A), highly conserved in 15 and 14 species, respectively (Figure S4). Two binding sites for miR-124 are also predicted (Figure 3A), the first conserved in 14 different species (Figure S4). Noteworthy, in long mammalian 3’ UTR, such as the 2.5 kb Nr3c2 3’ UTR, miRNA functional binding sites tend to cluster near the start or the end of the 3’ UTR [32].

To validate the functionality of these putative interactions, the complete mouse Nr3c2 3’ UTR was cloned dowstream of the firefly luciferase coding sequence (Nr3c2 3’ UTR) and this construct was transiently transfected into Hela cells along with miR-135a and miR-124 expression vectors (p135a and p124). These plasmids allow us to obtain high levels of U1 promoter-driven miR-135a and miR-124 expression, as shown by northern blot analysis (Figure S5). As presented in Figure 3B, we were able to demonstrate the predicted interactions between miR-135a and the 3’ UTR of Nr3c2 mRNA by means of approximately 50% of decrease in the expression of the Nr3c2 3’ UTR reporter upon miR-135a overexpression, comparing to the co-transfection of the reporter with the empty vector. The inhibition of luciferase expression is tightly dependent on the presence of a sequence in the Nr3c2 3’ UTR perfectly complementary to the miR-135a seed region. This was shown by transfection experiments with mutant reporter constructs containing the Nr3c2 3’ UTR mutated at the level of miR-135a seed binding sites, whose expression was unaffected by miRNA overexpression. However, the expression of Nr3c2 reporter was not altered by miR-124 overexpression (Figure 3B). A mutant reporter lacking the seed matches for miR-124 is unaffected by miR-124 overexpression, as expected, and is more than 60% inhibited by miR-135a overexpression. These reporter experiments indicate a direct functional interaction between miR-135a and the Nr3c2 3’ UTR and a no direct functional interaction between miR-124 and the Nr3c2 3’ UTR.

**Figure 4:**
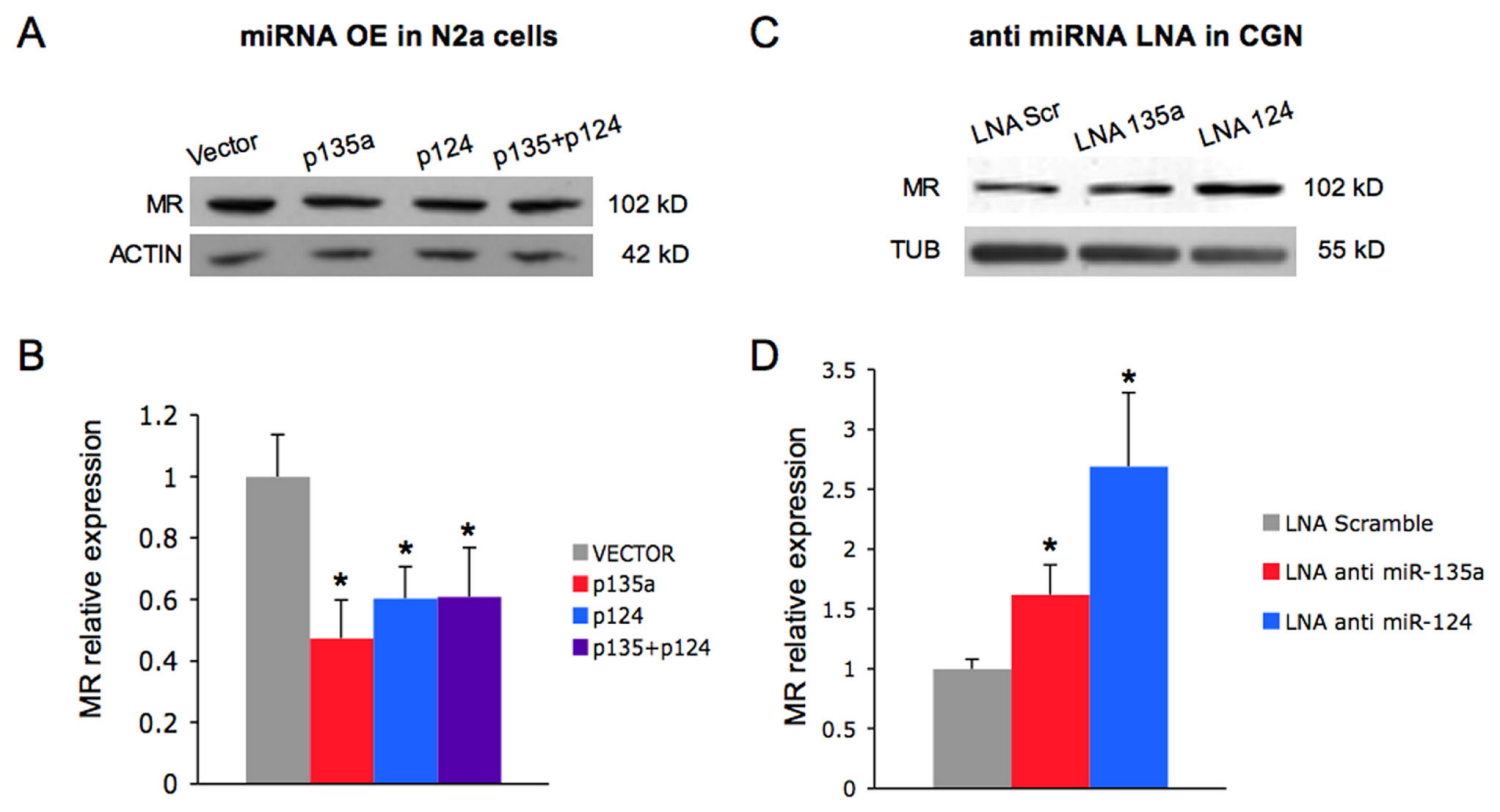
miR-135a and miR-124 regulate the expression of the endogenous MR protein. (A) N2a cells were transfected with miR-135a (p135a) and miR-124(p124) overexpressing vectors as indicated. p135a+p124 corresponds to the transfection with an equimolar mixture of the two vectors. A representative blot is shown. (B) Western blot analysis of endogenous MR expression was quantified by densitometry, normalized to actin as loading control and expressed relatively to empty vector transfected cells. Data represent the mean from three biological samples and three technical replicates ± SE. *P < 0.05, **P < 0.05 (pairwise Student’s *t*-test). (C) Cerebellar granule neurons (6+4 DIV) were transfected with LNA antisense oligonucleotides or scramble LNA as negative control. A representative blot is shown. (D) Western blot analysis of endogenous MR expression was quantified by densitometry, normalized to tubulin and expressed relatively to scramble LNA transfected cells. Data represent the mean from four biological samples and three technical replicates ± SE. *P < 0.05, (pairwise Student’s *t*-test).

To better examine the role of the predicted miRNAs in the control of Nr3c2 gene expression, we investigated whether the endogenous MR protein levels were affected by the modulation of candidate miRNAs. The mouse neuroblastoma cell line (N2a) and cerebellar granule neurons (CGN) express the MR at lower amounts than brain tissues, yet appreciable at both mRNA and protein levels (Figure S6). No expression of miR-135a and miR-124 was found in N2a cells (Figure S5, see empty vector lanes). Transfection of N2a cells with miR-135a expression constructs, resulting in an increased concentration of mature miRNAs (Figure S5), directed a significant reduction of MR protein expression (p135a, Figure 4A and B). As shown by quantitative analysis of western blots (Figure 4B), overexpression of mir-135a caused a 50% reduction of MR protein levels, compared to the transfection with the empty vector. Interestingly, the overexpression of mir-124 determined a 40% decrease on MR protein levels (Figure 4B). Moreover, we found a strong reduction of the MR expression after the combined overexpression of plasmids encoding for miR-135a and miR-124 (MR expression 60% of vector control transfection). These findings demonstrate that mir-135a and mir-124 are able to negatively affect the expression of the endogenous MR.

The ability of miR-135a to negatively regulate MR expressions was confirmed by overexpressing miR-135a from a different plasmid in which the miRNA transcription is driven by an H1-promoter (Figure S7A and B). Either the firefly reporter construct or the endogenous protein are significantly downregulated by miR-135a overexpression. Conversely to miRNA overexpression, the inhibition of endogenous miR-135a and miR-124 in CGN by LNA modified oligonucleotide transfections led to a statistically significant increase in the levels of endogenous MR protein (Figure 4C and D). Taken together these data suggest that mir-135a inhibits Nr3c2 mRNA translation by binding to either one or two sites present in the Nr3c2 3’ UTR; indirect effects of miR-124 on the MR endogenous protein expression have been observed both in N2a cells and CGN.

### Acute stress induces an increase in amygdala MR protein levels

**Figure 5:**
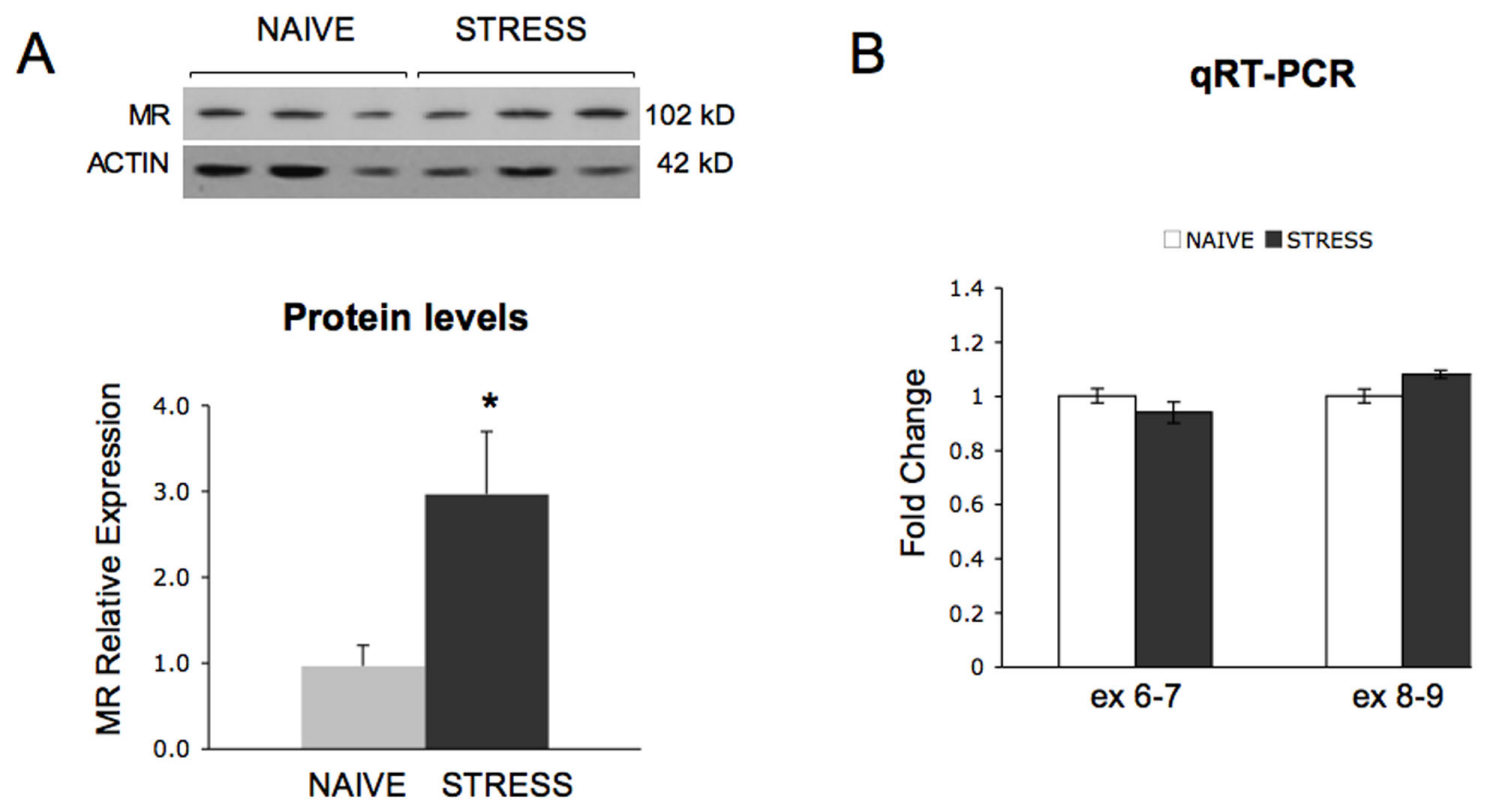
Acute stress increases MR protein levels in amygdala. (A) Steady state MR protein levels were measured by western blot analysis immediately after 2 hours of restraint. Lysates were obtained from pooled amygdala nuclei (*n*=4), 5 pools were generated from naive (*n*=20) and restrained mice (*n*=20). MR expression was normalized to actin signals in the same blot. Quantitative values are shown as mean ± SE *P < 0.05 (pairwise Student’s *t*-test). (B) qRT-PCR analysis of Nr3c2 transcript levels in naive and stressed mice (*n*=12 for both groups). Data are presented as mean ± SE. Ex 6-7 and exs 8-9 refer to the amplicons studied, corresponding to exons 6 and 7 (exs 6-7), and to exons 8 and 9 (exs 8-9) of the Nr3c2 coding sequence.

High levels of the Nr3c2 mRNA have been found in limbic regions such as the amygdala and the hippocampus [33] and high levels of MR protein have been described in the same brain regions [34]. To investigate whether two hours of restraint affect MR protein levels in the amygdala, we studied by quantitative western blot analysis the MR expression immediately after stress. As shown in Figure 5A acute stress induced a three-fold increase in the amygdala MR protein levels, this negatively parallels stress-induced alterations in miR-124 and miR-135a expression. Interestingly, no significant change was observed in Nr3c2 mRNA levels between stressed and naive mice (Figure 5B).

## Discussion

Multiple lines of evidence indicate that miRNAs play a key role in mediating cellular stress response [35]. To gain insight into the role of miRNAs in stress response, we analyzed stress-induced changes in miRNA expression in the amygdala after mouse restraint. Here we show that acute stress downregulates miR-135a and miR-124 expression in the amygdala. Moreover, we report that this effect parallels the increase in the MR expression level in the same brain region. Finally, we established miR-124 and miR-135a as regulators of the MR expression in mouse N2a cells and CGN.

The amygdala has a crucial role in the regulation of stress response [4,5], which makes this structure an interesting system to study how restraint stress modulates miRNA activities and to investigate miRNA role in the stress response. After two hours of stress we observed modifications on miRNA expression in the amygdala. This is consistent with the temporal profile of stress response in the brain, where after two hours of exposure to a stressor the rapid neurotransmitter activation and the corticosteroid dependent alteration of gene expression are already achieved [6]. In the present study we focused on the stress-induced downregulation of the brain-specific miR-135a and miR-124, and their possible role in the context of stress response. To test the hypothesis that these miRNAs directly participate in adaptive mechanisms by regulating the expression of components of stress response, we performed a computational analysis for predicted mRNAs targets. Among miR-135a predicted target genes we found as a top-score target Nr3c2, coding for the brain corticosteroid receptor MR. The MR, together with the glucocorticoid receptor (GR), is considered a master switch in the control of physiological and behavioral adaptation to stress [6,31,36]. On binding to the hormones, corticosteroid receptors translocate to the nucleus, where they act as modulators of gene expression by transactivation or transrepression [37,38]. However, recent evidence indicates that the initial stress reaction mediated by corticosteroids might be accomplished via limbic membrane MRs, activating non genomic signaling pathways [39,40].

We validated the predicted interaction between miR-135a and the mouse Nr3c2 3’ UTR by a luciferase assay, demonstrating a miR-135a induced reduction of Nr3c2 reporter expression. Furthermore, as shown by mutant constructs, miR-135a activity on Nr3c2 3’ UTR is direct and it is indeed strictly dependent on the integrity of the two cognate target sequences. In the Nr3c2 annotated 3’ UTR conserved sites for other miRNAs are present. Interestingly, a previous study aimed to the identification of miRNAs involved in kidney water–salt balance and blood pressure regulation, indicated the human NR3C2 gene as a potential target of miR-135a and miR-124 by mean of luciferase assays [41]. However, by luciferase experiments we were not able to confirm a direct interaction between miR-124 and the mouse Nr3c2 3’ UTR. Differences between mouse and human Nr3c2 3’ UTR sequences might account for the discrepancy between our and Sõber’s results. Nevertheless, the reporter assay may not be sufficient to predict the ability of a miRNA to modulate endogenous mRNA translation. Hence, we tested the validity of miR-135a and miR-124 binding sites, ascertained by reporter assays from our and others labs, by further experiments on mouse cells expressing the MR protein. We demonstrated that the overexpression in N2a cells of miR-135a and miR-124 determines a reduction of MR protein levels. It has been suggested that the number of target sites within a specific 3’ UTR can determine the degree of translational repression [42]. Indeed, we found that the transfection in neuroblastoma cells of miR-135a, that has two binding sites on the Nr3c2 3’ UTR, induces a strong suppression of the MR. In addition, knocking down endogenous miR-135a and miR-124 in CGN we were able to confirm the ability of both these miRNAs to affect the expression of the MR in a different cell system.

Other miRNAs are potential regulators of the Nr3c2 mRNA, suggestive of a complex regulatory network, as recently remarked [43]. Thus, it seems feasible that the MR gene expression is highly regulated in the amygdala at post-transcriptional level and, as previously discussed for many known target genes of miRNAs [44], multiple cis-regulatory sites in the Nr3c2 3’ UTR can be read by sets of coexpressed miRNAs. This complex scenario of the MR expression control might account for the indirect negative regulation of the MR expression by miR-124.

A broader effect of stress on miRNA expression in the brain is suggested by recent observations that indicate miRNA downregulation in the hippocampus and in the prefrontal cortex 24 hours after restraint [45,46]. These findings, together with our data, point out that stress-induced miRNA downregulation might involve different limbic structures and different temporal dynamics. Future studies will be required to better understand the spatial and temporal dynamic of stress induced modulation of miRNA expression. Interestingly, in *Aplysia* sensory neurons miR-124 expression was found to be regulated by a modulatory neurotransmission important for plasticity [47], suggesting a possible regulatory role of neurotransmission in stress-induced changes in miRNA expression.

Stressful stimuli activate the hypothalamic–pituitary–adrenal axis, leading to the secretion of adrenal stress hormones [1–3,48]. Subsequently, CORT feeds back on the brain binding to their cognate receptors, MR and GR. It has been demonstrated that an increase in the MR-mediated signaling in amygdala is neuroprotective, reducing both anxiety and CORT secretion [49]. Because of its high affinity for CORT, the MR is already heavily occupied by the basal levels of CORT [1]. Indeed, a raise in the MR protein levels was found after 24 hours of forced swimming in the hippocampus, neocortex, prefrontal cortex and amygdala [50]. However, to our knowledge little data is present in the literature regarding MR protein expression at early times after acute stress [51]. We show evidence of an induced expression of the MR protein in the amygdala immediately after 2 hours of restraint. Therefore, the observed MR protein increase, related to miR-135a and miR-124 down-regulation, might be instrumental for the MR functional activation in response to the stress-induced increase of CORT levels. Other factors, affecting positively the MR translational efficiency and protein stability, might cooperate with miRNAs to account for the three fold MR induction, detected at the protein level but not at the level of transcripts. The finding that the observed stress-induced increase in MR expression occurs exclusively at the protein level suggests a major contribution of mRNA translation control to the rapid changes of gene expression in the early phases of the stress response.

Importantly, miR-124 was recently identified as a regulator of GR expression [52]. Thus, the same miRNA, in concert with other miRNAs like miR-135a, might be responsible for the fine-tuning of corticosteroid receptors in order to maintain the correct MR/GR balance, necessary for effective coping with stress [6]. In a recent paper [53] mouse amygdala miR-34c was found to be induced 90 min after acute stress and the stress-related corticotropin releasing factor receptor type 1 was identified as one of the miR-34c targets. Therefore, increasing evidences indicate miRNAs as important mediators of early stress response in amygdala. The involvement of miRNAs in neuronal pathologies such as schizofrenia and depression has been recently shown [54,55]. It will be interesting to study whether miR-124 and miR-135a dysregulation is implicated in stress-related or major depressive disorders where a decreased expression of the amygdala MR was found [56].

In summary, we report that miR-135a and miR-124 are important components of the stress signaling response in the brain. They respond rapidly to stress and, exerting a control of the MR expression, might contribute to the post-transcriptional gene regulation of one of the key effectors of the corticosteroid cascade.

## Supporting Information

Figure S1 **Acute stress induces miR-135a and miR-124 downregulation in the amygdala.** Levels of mature miRNAs are quantified in the amygdala RNA pool by qRT-PCR using sno202 as internal control. The statistical test used for comparison was one-way ANOVA (*n*=9). Values are means ± SE *P <0.001 versus naive control mice.
(TIF)

Figure S2 **Expression of miR-135a in different brain structures.** Northern blot analysis of miR-135a expression in various mouse brain structures: Str, Striatum; Cb, Cerebellum; Ctx, Prefrontal cortex; OB, Olfactory bulb; Hippo, Hippocampus; Thal, Thalamus; Hypo, Hypotalamus; Amy, Amygdala; CGN, Cerebellar granule neurons in culture for 6 days (D6). U2 snRNA was used as an internal control.
(TIF)

Figure S3 **miR-135a target prediction.** Venn diagram of miR-135a target genes predicted by three independent algoritms, microT v 3.0, TargetScan 5.2, TargetScan 5.2. Numbers in non overlapping and overlapping areas indicate the number of genes identified by single or multiple algoritms. 38 are the target genes identified by the three algoritms.
(TIF)

Figure S4 **Sequence conservation of the miR-135a and miR-124 binding sites within the Nr3c2 3’ UTR.** miRNAs seeds and seed binding sequences in the 3’ UTR are indicated in blue. Free energies of the structures are reported.
(TIF)

Figure S5 **U1 promoter-driven miR-135a and miR-124 overexpression in Hela and N2a cells.** Nothern blot analysis of miRNA expression in Hela and N2a cells upon transient transfection of miRNA expression vectors (p135a, p135a-2, or p124) or empty vectors (pSP65). RNA samples have been prepared 24 hours after cell transfections. U2 snRNA was used as an internal control.
(TIF)

Figure S6 **MR expression in different tissues and cells.** (A) Quantification of Nr3c2 mRNA expression in tissue and cell lysates. The graph shows mRNA levels in amygdala (Amy), N2a cells (N2a) and cerebellar granule neurons (CGN), relatively to total brain mRNA levels. qRT-PCR analysis has been done using primers for exons 6 and 7 of the Nr3c2 coding sequence. The values, normalized for Actin, are means ± SE (n=6) (B) Immunoblot analysis of the MR expression in lysates from total brain (Brain), amygdala (Amy), N2a cells (N2a) and cerebellar granule neurons (CGN).
(TIF)

Figure S7 **MR down-regulation upon H1 promoter-driven expression of miR-135a.** (A) Nr3c2 luciferase reporter or miR-135a sensor constructs were co-transfected into Hela cells together with empty vector or mir-135a expression vector (ps135a). Luciferase activity was measured 24 hours post-transfection. Values are expressed relatively to the internal renilla luciferase activity and presented as percentage of activity achieved in the presence of the empty control vector. Results are shown as means ± SE (n=6). **P<0.005 (pairwise Student’s *t*-test). (B) Representative western blots of lysates from cells transfected with empty vector and miR-135a expression vectors (ps135a).
(TIF)

Methods S1 **Supplementary methods.**
(DOC)

Table S1 **Oligonucleotides used in this study.**
(DOC)
