# Supplementary figures and images for: Acute Stress Alters Amygdala microRNA miR-135a and miR-124 Expression: Inferences for Corticosteroid Dependent Stress Response
Source: PLoS One. 2013 Sep 4;8(9):e73385. doi: 10.1371/journal.pone.0073385 (PMC3762773; doi:10.1371/journal.pone.0073385)

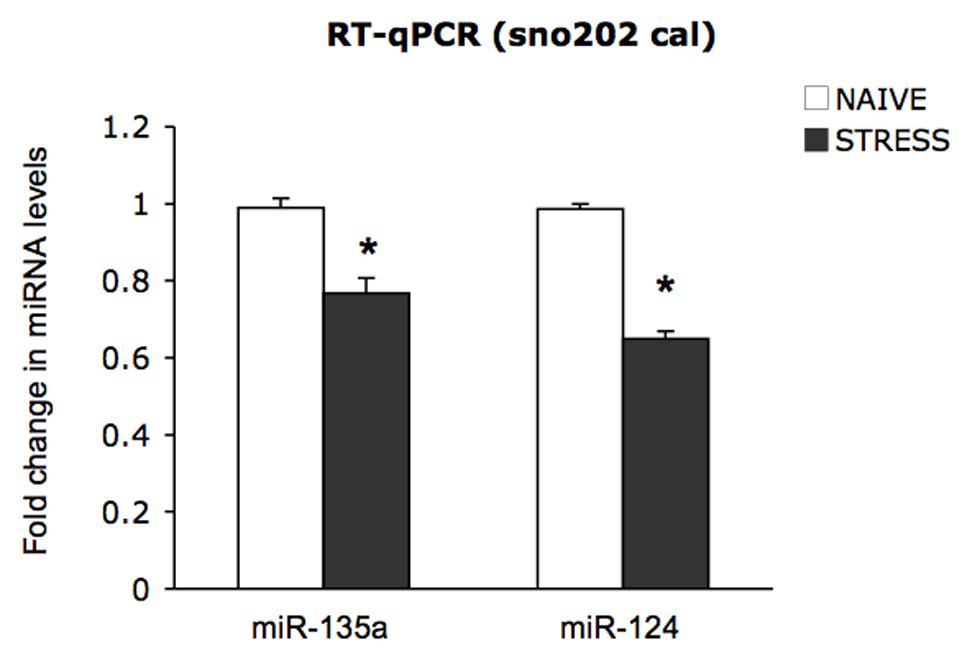

Supplement: Figure S1 — Acute stress induces miR-135a and miR-124 downregulation in the amygdala. Levels of mature miRNAs are quantified in the amygdala RNA pool by qRT-PCR using sno202 as internal control. The statistical test used for comparison was one-way ANOVA (n=9). Values are means ± SE *P <0.001 versus naive control mice. (TIF) [file pone.0073385.s001.tif]

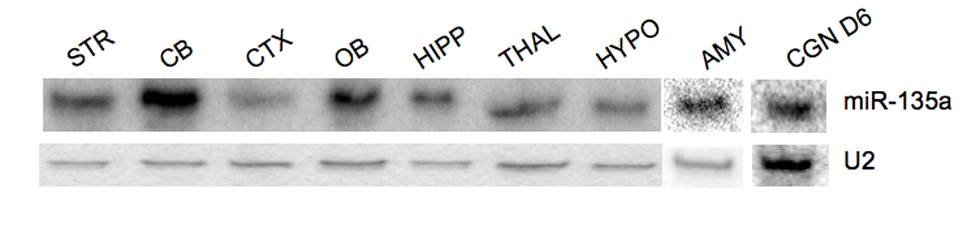

Supplement: Figure S2 — Expression of miR-135a in different brain structures. Northern blot analysis of miR-135a expression in various mouse brain structures: Str, Striatum; Cb, Cerebellum; Ctx, Prefrontal cortex; OB, Olfactory bulb; Hippo, Hippocampus; Thal, Thalamus; Hypo, Hypotalamus; Amy, Amygdala; CGN, Cerebellar granule neurons in culture for 6 days (D6). U2 snRNA was used as an internal control. (TIF) [file pone.0073385.s002.tif]

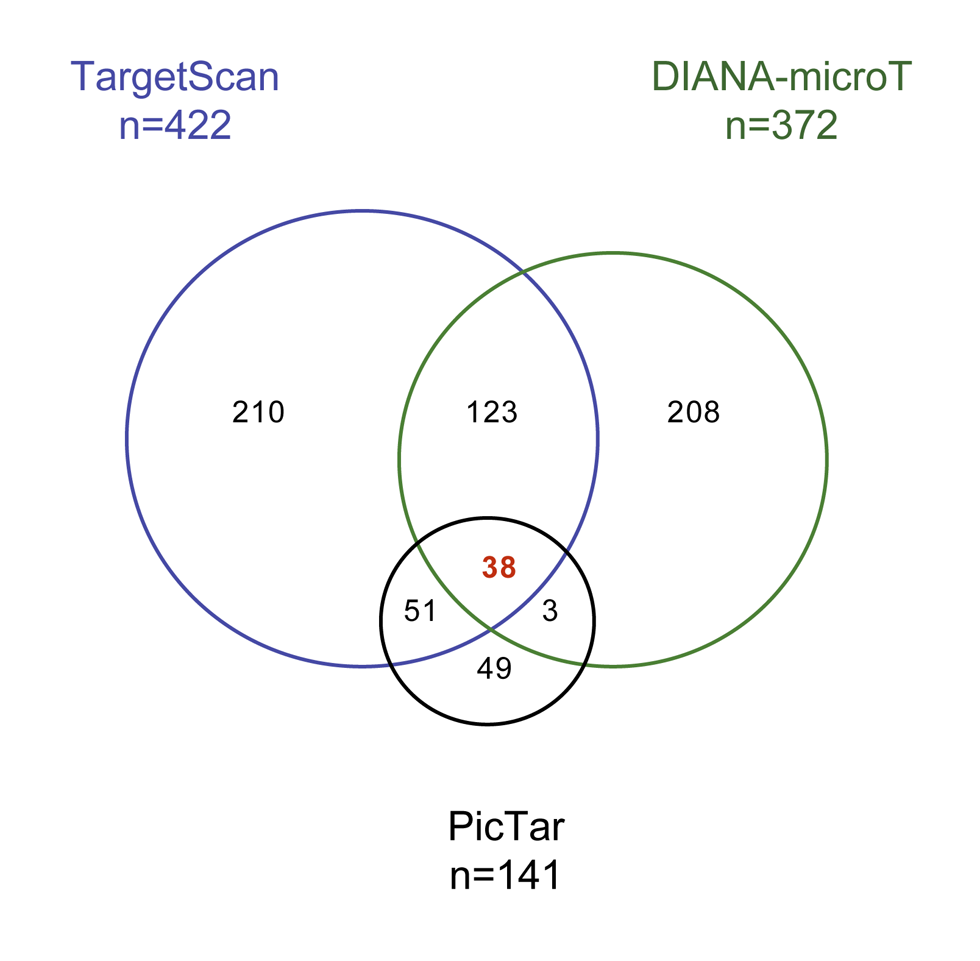

Supplement: Figure S3 — miR-135a target prediction. Venn diagram of miR-135a target genes predicted by three independent algoritms, microT v 3.0, TargetScan 5.2, TargetScan 5.2. Numbers in non overlapping and overlapping areas indicate the number of genes identified by single or multiple algoritms. 38 are the target genes identified by the three algoritms. (TIF) [file pone.0073385.s003.tif]

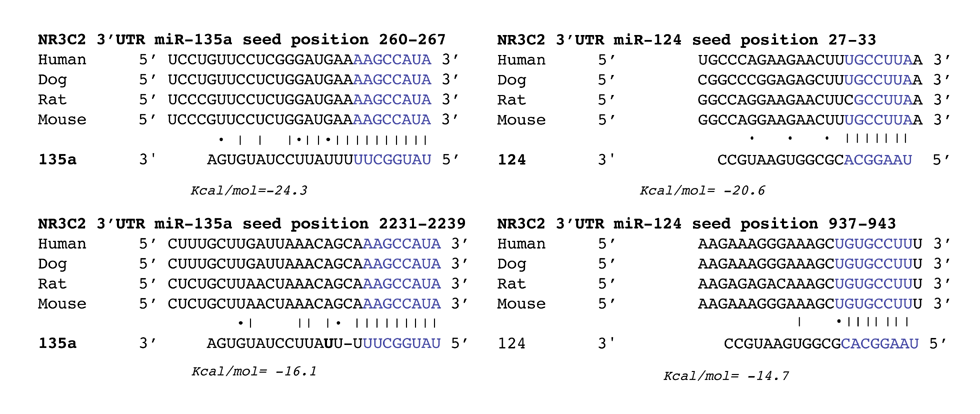

Supplement: Figure S4 — Sequence conservation of the miR-135a and miR-124 binding sites within the Nr3c2 3’ UTR. miRNAs seeds and seed binding sequences in the 3’ UTR are indicated in blue. Free energies of the structures are reported. (TIF) [file pone.0073385.s004.tif]

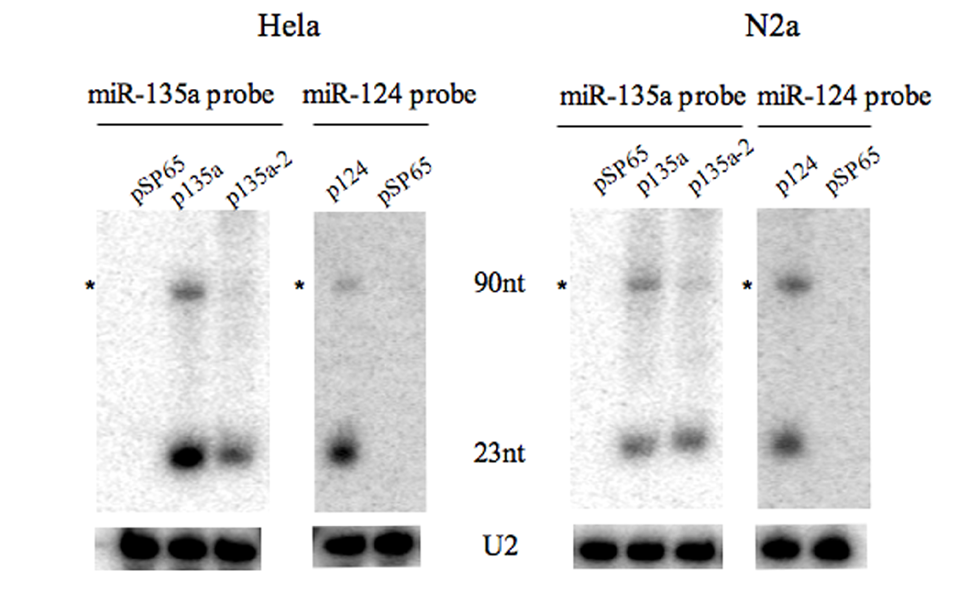

Supplement: Figure S5 — U1 promoter-driven miR-135a and miR-124 overexpression in Hela and N2a cells. Nothern blot analysis of miRNA expression in Hela and N2a cells upon transient transfection of miRNA expression vectors (p135a, p135a-2, or p124) or empty vectors (pSP65). RNA samples have been prepared 24 hours after cell transfections. U2 snRNA was used as an internal control. (TIF) [file pone.0073385.s005.tif]

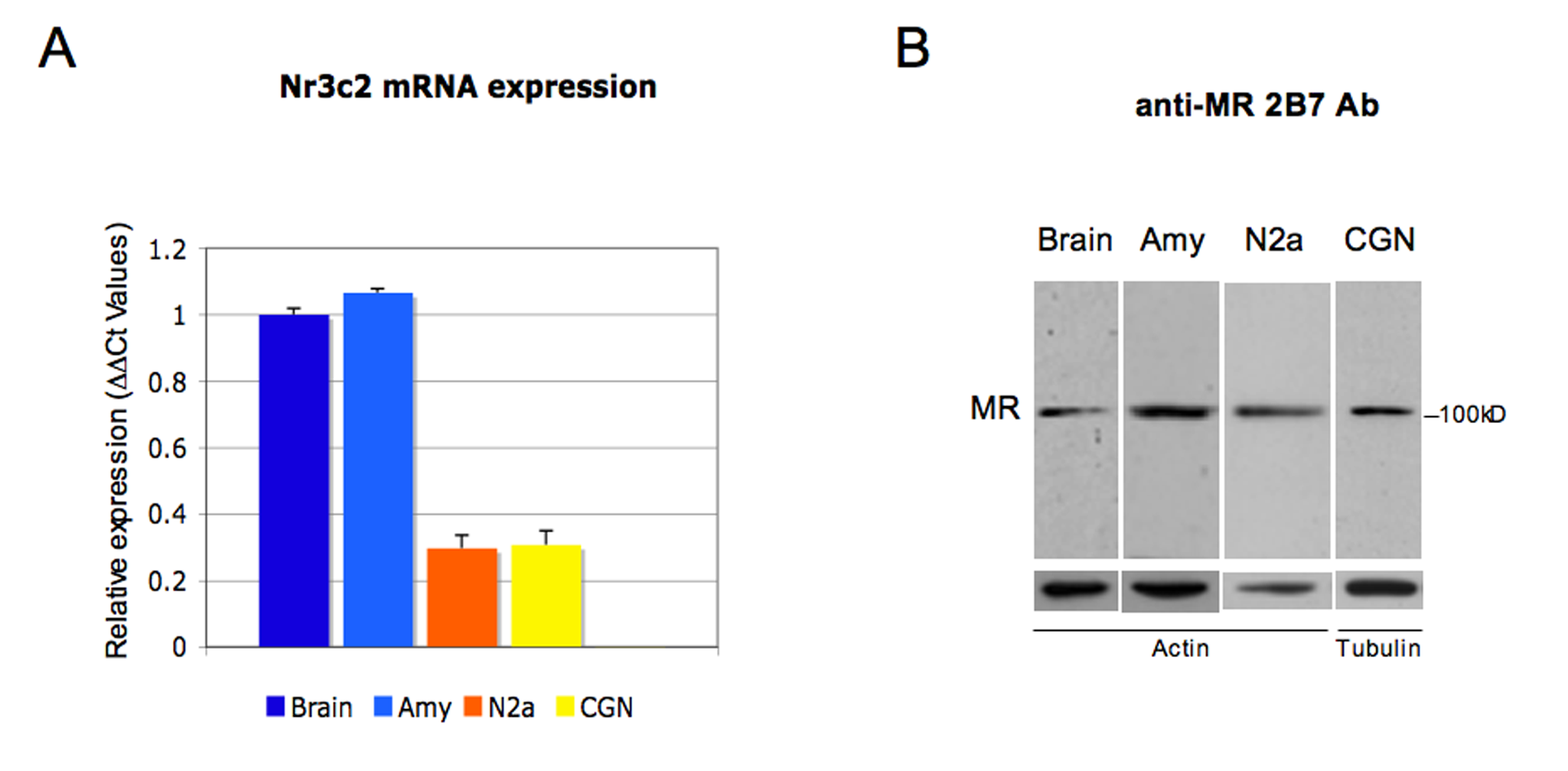

Supplement: Figure S6 — MR expression in different tissues and cells. (A) Quantification of Nr3c2 mRNA expression in tissue and cell lysates. The graph shows mRNA levels in amygdala (Amy), N2a cells (N2a) and cerebellar granule neurons (CGN), relatively to total brain mRNA levels. qRT-PCR analysis has been done using primers for exons 6 and 7 of the Nr3c2 coding sequence. The values, normalized for Actin, are means ± SE (n=6) (B) Immunoblot analysis of the MR expression in lysates from total brain (Brain), amygdala (Amy), N2a cells (N2a) and cerebellar granule neurons (CGN). (TIF) [file pone.0073385.s006.tif]

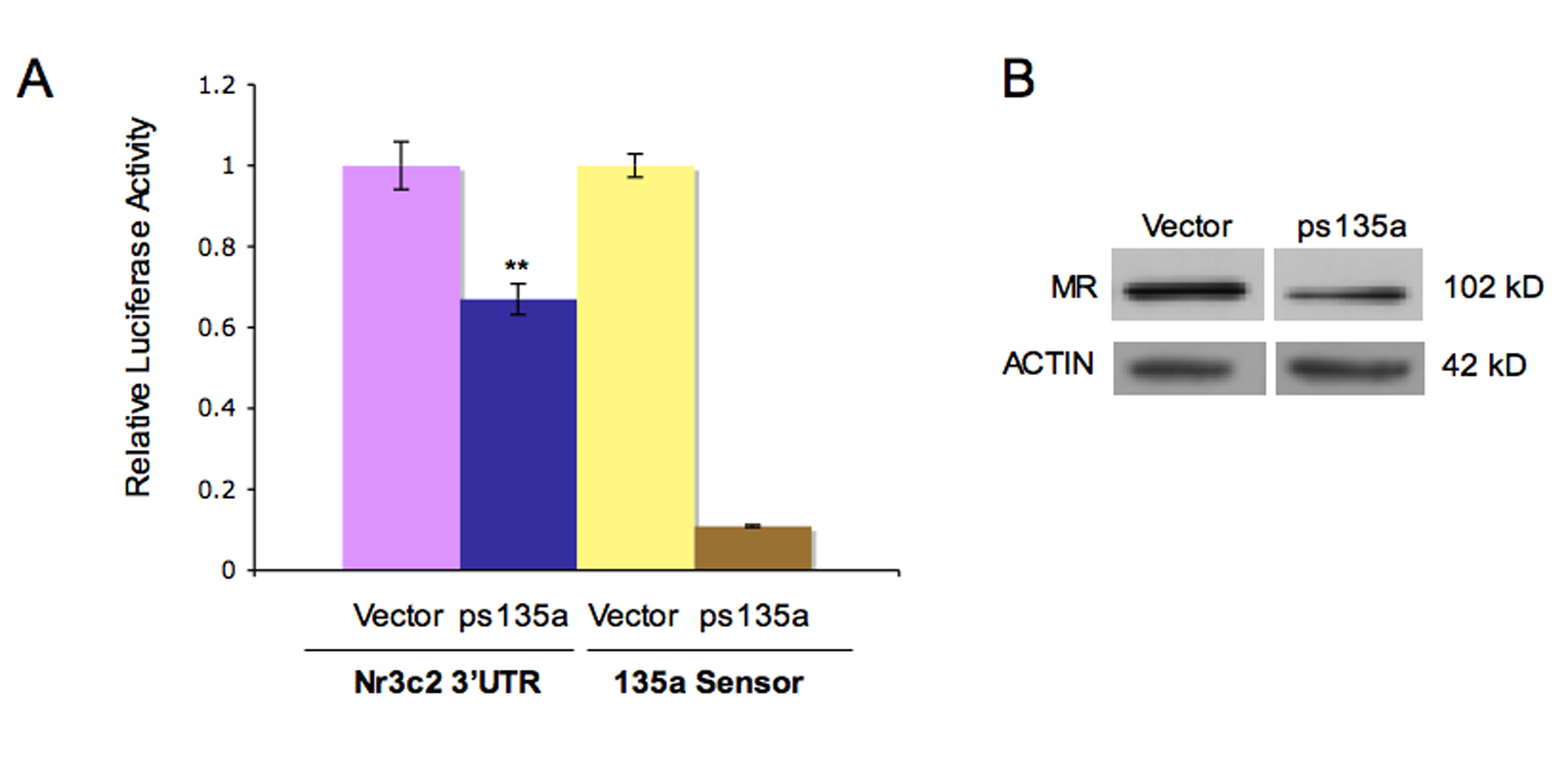

Supplement: Figure S7 — MR down-regulation upon H1 promoter-driven expression of miR-135a. (A) Nr3c2 luciferase reporter or miR-135a sensor constructs were co-transfected into Hela cells together with empty vector or mir-135a expression vector (ps135a). Luciferase activity was measured 24 hours post-transfection. Values are expressed relatively to the internal renilla luciferase activity and presented as percentage of activity achieved in the presence of the empty control vector. Results are shown as means ± SE (n=6). **P<0.005 (pairwise Student’s t-test). (B) Representative western blots of lysates from cells transfected with empty vector and miR-135a expression vectors (ps135a). (TIF) [file pone.0073385.s007.tif]
