# Supplementary material for: Acute Stress Alters Amygdala microRNA miR-135a and miR-124 Expression: Inferences for Corticosteroid Dependent Stress Response
Source: PLoS One. 2013 Sep 4;8(9):e73385. doi: 10.1371/journal.pone.0073385 (PMC3762773; doi:10.1371/journal.pone.0073385)
Supplement: Methods S1 — Supplementary methods. (DOC) [file pone.0073385.s008.doc]

**Methods S1. Supplementary Methods**

**DNA constructs**

Plasmid ps135a was generated by cloning into BglII/ HindIII sites of the pSuper vector (Oligoengine) a 60-bp hairpin sequence containing the miR-135a mature sequence. The 135a Sensor plasmid was generated by cloning into the XbaI site of the pGL3 control vector an oligonucleotide antisense to the miR135a sequence.

**Northern blot analysis**

For northern blots 4 µg of total RNA, isolated from amygdala nuclei, or 10 µg of total RNA from other brain tissues and cells, were electrophoretically separated on 15% polyacrilamide/TBE/urea gels and electroblotted in 0.5x TBE buffer to Hybond N^+^ membranes (Amersham), using a wet transfer apparatus (Hoefer). After UV cross-linking of the RNA to the membrane, hybridization was performed overnight in 7% SDS, 0.2 M Na_2_PO_4_, pH 7.2. Probes for the snRNA U2 and miR-124 were labeled with [γ-^32^P]-ATP (Amersham Biosciences) by T4 polynucleotide kinase (Fermentas, Thermo Scientific). StarFire high specific activity probes for miR-135a were prepared as described (Behlke et al, 2000) by incorporation of a [α-^32^P]dATP. After washing, the membranes were imaged and quantified using the Typhoon 8600 system (Amersham). U2 was used as internal control.

**Supplemetary reference**

Behlke MA, Dames SA, McDonald WH, Gould KL, Devor EJ, et al (2000) Use of high specific activity StarFire oligonucleotide probes to visualize low-abundance pre-mRNA splicing intermediates in S. pombe. Biotechniques 29: 892-897.
