# Supplementary material for: Acute Stress Alters Amygdala microRNA miR-135a and miR-124 Expression: Inferences for Corticosteroid Dependent Stress Response
Source: PLoS One. 2013 Sep 4;8(9):e73385. doi: 10.1371/journal.pone.0073385 (PMC3762773; doi:10.1371/journal.pone.0073385)
Supplement: Table S1 — Oligonucleotides used in this study. (DOC) [file pone.0073385.s009.doc]

**Table S1. Oligonucleotides used in this study**

**Cloning and sequencing**

***pGL3 constructs***

*Nr3c2 3’UTR*

NR3C2 fwd/XbaI: 5’-GCTCTAGATGGAAGATGCCGGCCAG -3’

NR3C2 rev/XbaI: 5’-GCTCTAGAGTAATCTGCTTACAGTCCTTTGCAA -3’

NR3C2 fwd1: 5’-GCTTCTGCTTCTTCCTGC-3’

NR3C2 rev1: 5’-GCAGGAAGAAGCAGAAGC-3’

NR3C2 fwd2: 5’-GCCTTAAGTTTCCCCACGTT-3’

NR3C2 rev2: 5’-GGACAGGCATTCAACAGTGA-3’

NR3C2 rev3: 5’-CATGCAGGGCATCTCACTTA-3’

*Nr3c2 m135a 3’UTR*

NR2C3 135mut1 fwd: 5’-CTCTGGATGAAAA**TGA**ATATCTAGTCAATAAC -3’

NR2C3 135mut1 rev: 5’-GTTATTGACTAGATAT**TCA**TTTTCATCCAGAG -3’

NR2C3 135mut2 fwd: 5’- CTAAACAGCAAA**TGA**ATATTCTCTAAAATTC -3’

NR2C3 135mut2 rev: 5’- GAATTTTAGAGAATAT**TCA**TTTGCTGTTTAG -3’

*Nr3c2 m124 3’UTR*

NR2C3 124mut1 fwd/XbaI: 5’-GCTCTAGATGGAAGATGCCGGCCAGGAAGAACTTTTGATTAAGTTTCC -3’

NR2C3 124mut1 rev: 5’- GCTAAAAAAA**TCA**ACAGCTTTCCCTTTCTTAAATACAC -3’

NR2C3 124mut2 fwd: 5’- GGGAAAGCTGT**TGA**TTTTTTTAGC -3’

*135a Sensor*

135a Sensor fwd/XbaI: 5’-GCTCTAGATGGTAGGGTATATTCACATAGGAATAAAAAGCCATGTCCCTTTCCATTGCTCTAGAGC-3’

135a Sensor rev/XbaI: 5’-GCTCTAGAGCAATGGAAAGGGACATGGCTTTTTATTCCTATGTGAATATACCCTACCATCTAGAGC-3’

***pSuper constructs***

p135a fwd/BglII: 5’-GATCCCCTCACATAGGAATAAAAAGCCATATTCAAGAGATATGGCTTTTTATTCCTATGTGATTTTTA-3’

p135a rev/HindIII: 5’-AGCTTAAAAATCACATAGGAATAAAAAGCCATATCTCTTGAATATGGCTTTTTATTCCTATGTGAGGG-3’

***pSP65 constructs***

ps135a-1 fwd/BglII: 5’- GAAGATCTTAGACTGTCCAGAGCCCAGTGG -3’

ps135a-1 rev/KpnI: 5’-GGGTACCTCACCCCATCCTGTCATAGCTG-3’

**qRT-PCR**

*NR3C2 ex 6-7*

Nr3c2 Ex6 fwd: 5’-ATCATCGTTTGCCTTGAGTTG-3’

Nr3c2 Ex6-7 rev: 5’-ACTGATGCATCTTCTCTTCATTAAAAA-3’

*NR3C2 ex 8-9*

NR3C2 Ex8-9 fwd: 5’-CTCCATGCATGATTTGGTGAA-3’

Nr3c2 Ex9 rev: 5’-TGGTCGCTGATGATCTCCAC-3’

*Actin*

ACTIN fwd: 5’-TGTGACGTTGACATCCGTAAAGA-3’

ACTIN rev: 5’-CTGCATCCTGTCAGCAATGC-3’

**Northern blot**

as 135aSF: 5’-TCACATAGGAATAAAAAGCCATACCATCC-3’

SF template: 5’-TTTTTTTTTTGGATGG-3’

as U2: 5’-TTAGCCAAAAGGCCGAGAAGC-3’

as 124: 5’-ATCAAGGTCCGCTGTGAACACG-3’
